# Identification of immune and Toll-like receptor signaling pathway related feature lncRNAs to construct diagnostic nomograms for acute ischemic stroke

**DOI:** 10.1038/s41598-023-33059-5

**Published:** 2023-04-20

**Authors:** Zhuo-Yi Su, Zi-Qiao Yu, Bo Yao, De-Xi Zhao

**Affiliations:** 1 Changchun University of Chinese Medicine, No.1035 Boshuo Road, Jing Yue National High-Tech Industrial Development Zone, Changchun, 130117 China; 2 School of Aeronautical Fundamentals, Aviation University of Air Force, Changchun, 130041 China

**Keywords:** Computational biology and bioinformatics, Software

## Abstract

We aimed to identify the immune and Toll-like receptor (TLR) signaling pathway related feature lncRNAs to construct the diagnostic nomograms for acute ischemic stroke (AIS). Two AIS-associated expression profiles GSE16561 and GSE22255 were downloaded from NCBI Gene Expression Omnibus, the former was the training set and the latter was the validation set. The differential expression genes (DEGs) and lncRNAs (DElncRNAs) related to TLR signaling pathway were identified between AIS and control groups. The single sample gene set enrichment analysis (ssGSEA) was applied to evaluate the immune infiltration. The immune and TLR signaling pathway related DElncRNAs were determined. Three optimization algorithms were utilized to select the immune and TLR signaling pathway related feature lncRNAs to construct the diagnostic nomograms of AIS. Based on the lncRNA signature, a ceRNA network was constructed. 37 DEGs and 28 DElncRNAs related to TLR signaling pathway were identified in GSE16561. 16 immune cell types exhibited significant differences in distribution between AIS and control groups. 28 immune and TLR signaling pathway related DElncRNAs were determined. 8 immune and TLR signaling pathway related feature lncRNAs were selected. The diagnostic nomograms of AIS performed well in both datasets. A ceRNA network was constructed consisting of 7 immune and TLR signaling pathway related feature lncRNAs as well as 19 AIS related miRNAs and 21 TLR signaling pathway related genes. LINC00173, LINC01089, LINC02210, MIR600HG, SNHG14, TP73-AS1, LINC00680 and CASC2 may be the potential biomarkers of AIS diagnosis, and TLR signaling pathway may be a promising immune related therapeutic target for AIS.

## Introduction

Stroke is one of the leading causes of death and disability globally, most of which are ischemic^1^. Acute ischemic stroke (AIS) is defined by the sudden interruption of focal cerebral blood flow caused by thrombosis or embolism^2^. Currently, the therapeutic options of AIS are limited, and intravenous thrombolysis and endovascular thrombectomy are available for the appropriate patients^3^. However, aiming at restoring cerebral blood flow, both approaches are time-critical, and the narrow therapeutic time windows limit their widespread applications among patients, prompting the demand and development of novel therapeutic strategies that target the underlying mechanisms of cellular damage and recovery in the brain and periphery, such as the immune response^4,5^. The immune system is involved in the occurrence and progression of AIS, and plays a multifaceted role in the disease^6^. The Toll-like receptor (TLR) signaling pathway is the major innate immune response mechanism that mediates cerebral ischemic injury^7^. Activation of TLRs signal after ischemic insults triggers the initiation of downstream inflammatory cascades and the production of cytokines and chemokines, and further induces the immune responses, which could be either neuroprotective or detrimental to AIS^8^. Therefore, considering the important role of TLR signaling pathway in the pathogenesis of AIS, it may be a promising avenue for future immunotherapy of a large spectrum of AIS patients.

Studies have reported that long non-coding RNAs (lncRNAs) were differentially expressed in ischemic stroke patients and post-ischemic mouse models to modulate the downstream gene transcription in response to ischemic injury^9,10^. LncRNAs participate in the development of ischemic stroke through various signaling pathways and biological processes following cerebral ischemic injury, including immune response, inflammation, cell apoptosis, autophagy and angiogenesis, suggesting that lncRNAs might hold a potential to be the novel biomarkers and targets in the diagnosis and treatment for AIS, and elucidating the biological roles and molecular regulation mechanisms of lncRNAs involved in the pathogenesis of AIS could contribute to the management and therapeutics for AIS patients^11,12^.

Herein, based on expression profiles from AIS patients, we identified the immune and TLR signaling pathway related lncRNAs through ssGSEA, and then different optimization algorithms were applied to further select feature lncRNAs. Using the lncRNA signature, the diagnostic nomograms were constructed with good performance. Combining immune and TLR signaling pathway related feature lncRNAs as well as AIS related miRNAs and TLR signaling pathway related genes, a ceRNA network was constructed which improved the knowledge of how these feature lncRNAs act in the TLR signaling pathway. The immune and TLR signaling pathway related feature lncRNAs may be the candidate biomarkers of AIS diagnosis, providing the potential for TLR signaling pathway as the promising immune related therapeutic target for AIS.

## Methods

### Data collection and preprocessing

Two AIS-associated expression profiles GSE16561 and GSE22255 were downloaded from NCBI Gene Expression Omnibus (http://www.ncbi.nlm.nih.gov/geo/), which provides an invaluable resource of publicly available gene expression data. GSE16561 was served as the training set, while GSE22255 was the validation set. In GSE16561, peripheral whole blood samples were obtained from 39 AIS patients and 24 healthy controls, and the platform was GPL6883 Illumina HumanRef-8 v3.0 expression beadchip. In GSE22255, whole blood samples were collected from 20 cases and 20 controls, and the platform was GPL570 Affymetrix Human Genome U133 Plus 2.0 Array. The detailed annotation files of two platforms were downloaded from Ensembl (http://asia.ensembl.org/index.html) to re-annotate the array data to gain the expression levels of mRNAs and lncRNAs.

### Identification of TLR signaling pathway related lncRNAs

The Molecular Signatures Database (MSigDB) is a collection of the annotated gene sets that are divided into 9 major collections. KEGG subset of Canonical Pathways in C2 collection (curated gene sets) was downloaded from MsigDB (http://www.gsea-msigdb.org/gsea/downloads.jsp), which contained the information of 186 KEGG pathways and the corresponding gene sets (Supplementary Table S1). The TLR signaling pathway was singled out, and there were 102 genes in the gene set. The expression of 102 TLR signaling pathway related genes was extracted from GSE16561 dataset, and then the differential expression genes (DEGs) associated with TLR signaling pathway between AIS patients and controls were identified using limma package in R3.6.1. The thresholds were set as |fold change| (|FC|) > 1.2 and false discovery rate (FDR) < 0.05. The differential expression lncRNAs (DElncRNAs) in GSE16561 were determined in the same way. The Pearson correlation coefficients (PCCs) between the expressions of DEGs associated with TLR signaling pathway and DElncRNAs were calculated by cor function in R3.6.1 in order to measure the co-expression of DEGs and DElncRNAs. The DEG-DElncRNA pairs with |PCC|> 0.3 and p < 0.05 were regarded as the significant co-expression pairs to construct a co-expression network, which was visualized by Cytoscape3.6.1 (https://cytoscape.org/). The lncRNAs involved in the significant DEG-DElncRNA co-expression pairs were considered to be associated with TLR signaling pathway.

### Identification of immune and TLR signaling pathway related lncRNAs

Based on the single sample gene set enrichment analysis (ssGSEA), GSVA package in R3.6.1 was applied to assess the immune infiltration of samples in GSE16561. 28 immune cell types and their marker genes were obtained from Bindea et al.^13^. The t-test was applied to detect the differences of immune cell types in distribution between AIS and control groups, and the threshold was p < 0.05. The PCCs between lncRNAs associated with TLR signaling pathway and immune cell types with significant differences in distribution between groups were calculated by cor function in R3.6.1. The lncRNAs in the significant correlations with |PCC|> 0.3 and p < 0.05 were regarded as the immune and TLR signaling pathway related lncRNAs.

### Optimization of the lncRNA signature and construction of the diagnostic nomogram

In the training set GSE16561, three optimization algorithms were applied for the feature lncRNA selection, including least absolute shrinkage and selection operator (LASSO), recursive feature elimination (RFE) and random forest (RF), which were implemented by lars package, caret package and randomForest package in R3.6.1, respectively. The intersection of three feature lncRNA sets was served as the immune and TLR signaling pathway related lncRNA signature.

The nomogram was constructed by rms package in R3.6.1, and then decision curve analysis was performed by rmda package in R3.6.1 to evaluate the net benefit of the nomogram. The nomogram was also built in the validation set GSE22255 to verify the efficiency of the diagnostic model.

### Construction of a ceRNA network

The AIS-associated miRNAs were downloaded from the Human microRNA Disease Database (HMDD) v3.2 (http://www.cuilab.cn/hmdd) that experimentally supported human miRNA and disease associations^14^. Then, associations between the immune and TLR signaling pathway related feature lncRNAs and miRNAs associated with AIS were explored using DIANA-LncBasev2 (http://carolina.imis.athena-innovation.gr/diana_tools/web/index.php?r=lncbasev2%2Findex-experimental), which provided experimentally supported and in silico predicted miRNA recognition elements (MREs) on lncRNAs^15^. The threshold was set as score > 0.7. Next, on this basis, the mRNA targets of the AIS-associated miRNAs were searched in the starBase v2.0 (http://starbase.sysu.edu.cn/)^16^. Genes that were both mRNA targets and DEGs associated with TLR signaling pathway were reserved. Finally, a ceRNA network was constructed by combining the lncRNA-miRNA interactions (immune and TLR signaling pathway related feature lncRNAs—miRNAs associated with AIS) and miRNA-mRNA interactions (miRNAs associated with AIS—DEGs associated with TLR signaling pathway), which was visualized by Cytoscape3.6.1.

## Results

### TLR signaling pathway related lncRNAs

**Figure 1:**
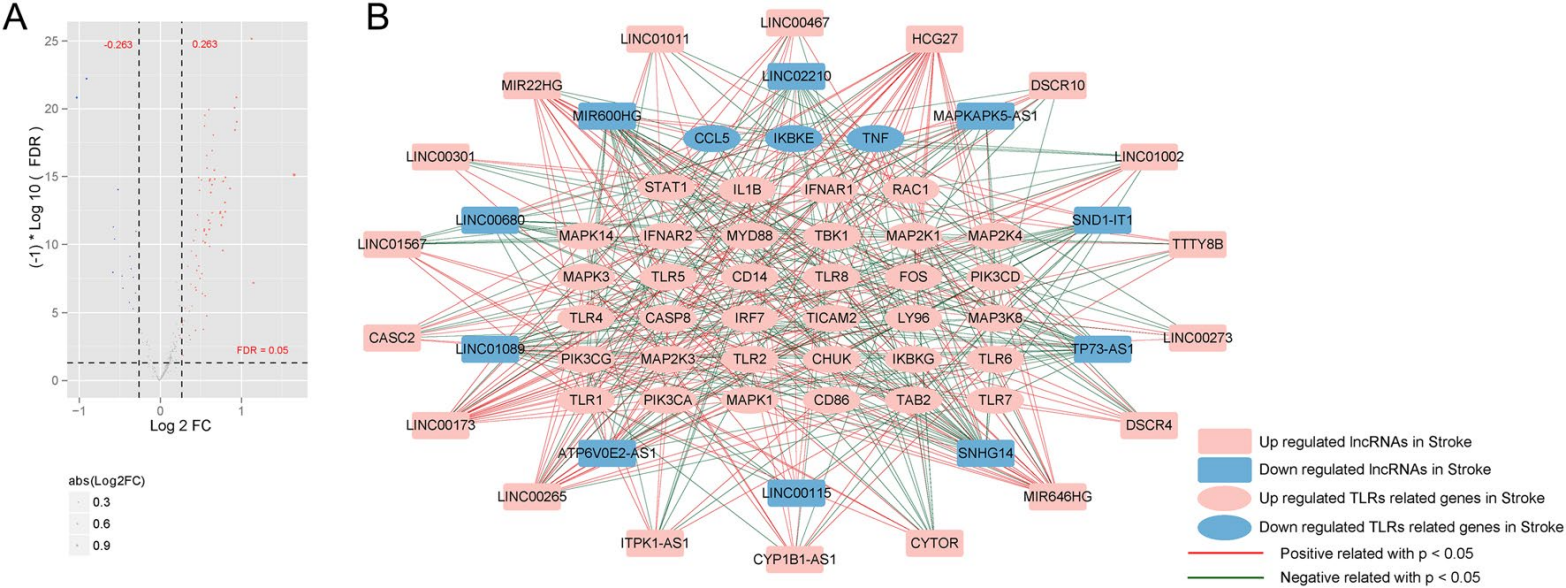
Identification of the DEGs associated with TLR signaling pathway and DElncRNAs (**A**) and their co-expression network (**B**).

The expression of lncRNAs and 102 TLR signaling pathway related genes was extracted from GSE16561. A total of 28 DElncRNAs (18 up-regulated and 10 down-regulated) and 37 DEGs associated with TLR signaling pathway (34 up-regulated and 3 down-regulated) were identified (Fig. 1A and Supplementary Table S2). Based on the PCCs between the DElncRNAs and DEGs, 436 significant co-expression DEG-DElncRNA pairs were determined (Supplementary Table S3), including 28 DElncRNAs and 37 DEGs associated with TLR signaling pathway. Figure 1B depicts the co-expression network, and 28 lncRNAs in the network were considered to be associated with TLR signaling pathway.

### Immune and TLR signaling pathway related lncRNAs

**Figure 2:**
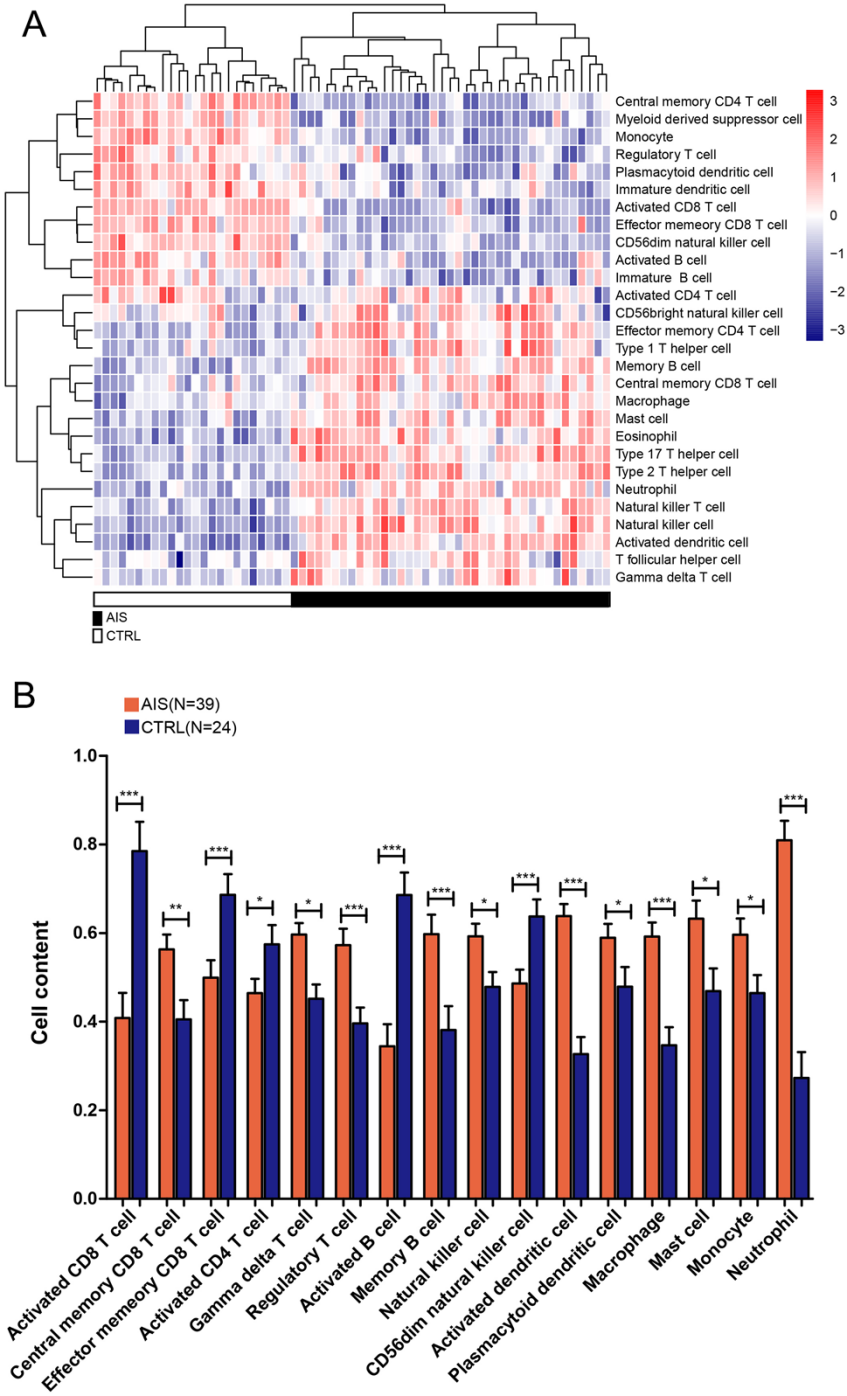
The immune landscape of samples in GSE16561. (**A**) Heatmap of immune cell proportion distribution of samples between AIS and control groups, implemented by by GSVA package in R3.6.1 (http://www.bioconductor.org/packages/release/bioc/html/GSVA.html). (**B**) Bar chart of immune cell types with significant differences in distribution between AIS and control groups.

**Figure 3:**
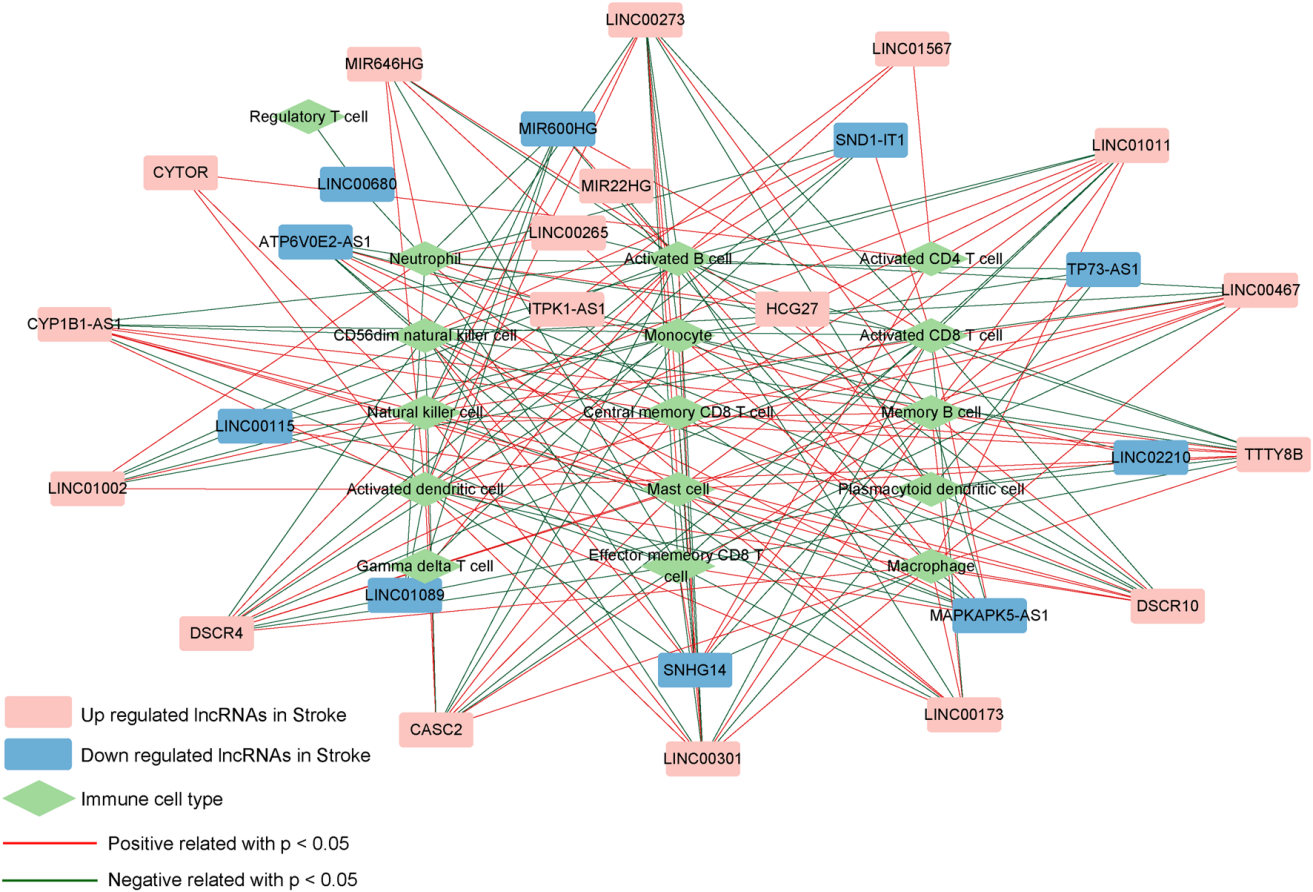
The lncRNA-immune cell correlation network consisting of TLR signaling pathway related lncRNAs and immune cell types with significant differences in distribution.

The immune infiltration of samples in GSE16561 was assessed by the ssGSEA method (Fig. 2A), and 16 immune cell types exhibited significant differences in distribution between AIS and control groups (Fig. 2B). Based on the PCCs between 28 lncRNAs associated with TLR signaling pathway and 16 immune cell types, 195 significant lncRNA-immune cell correlations were determined (Supplementary Table S4). Figure 3 shows the lncRNA-immune cell correlation network, and 28 lncRNAs in the network were considered to be the immune and TLR signaling pathway related lncRNAs.

### The diagnostic nomogram

LASSO, RFE and RF algorithms were applied to optimize the immune and TLR signaling pathway related lncRNAs, which screened 15, 8 and 28 feature lncRNAs, respectively (Supplementary Table S5). Through the comparison of three optimized lncRNA sets, a combination of 8 common feature lncRNAs among three sets was regarded as the immune and TLR signaling pathway related lncRNA signature, including LINC00173, LINC01089, LINC02210, MIR600HG, SNHG14, TP73-AS1, LINC00680 and CASC2.

**Figure 4:**
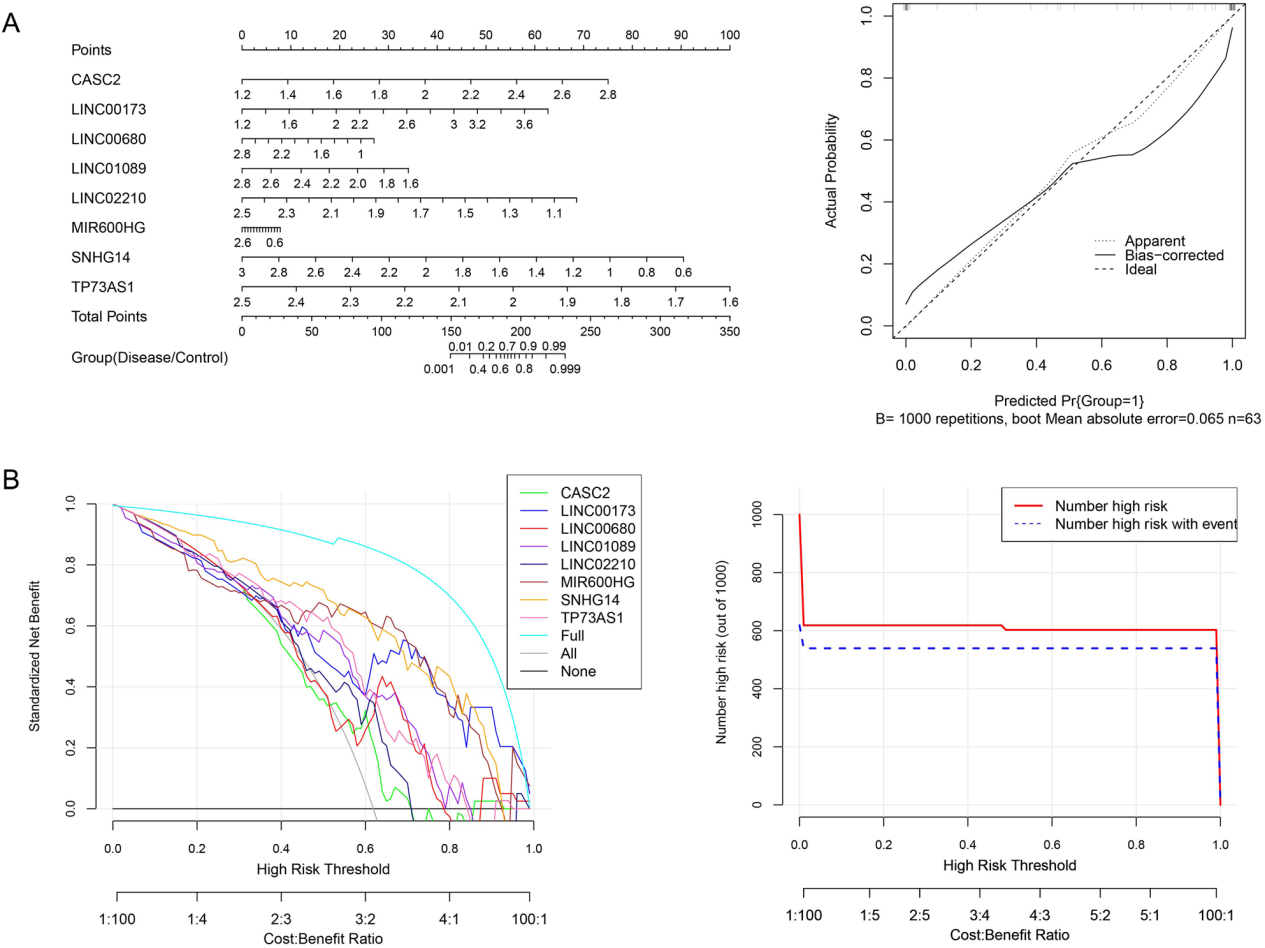
The diagnostic nomogram applied to the training set GSE16561 and the corresponding calibration curve (**A**) and the decision curve analysis (**B**).

Based on the lncRNA signature, a nomogram was constructed in the training set GSE16561, and the calibration curve for the diagnosis of disease demonstrated a good agreement between the nomogram and actual observation (Fig. 4A). The DCA depicted that the nomogram yielded the highest net benefit among the whole range of threshold probabilities (Fig. 4B). In the validation set GSE22255, the nomogram possessed the satisfactory diagnostic ability as well (Supplementary Fig. S1). The eight immune and TLR signaling pathway related feature lncRNAs exhibited the consistent differential expression patterns between groups in both datasets, in which CASC2 and LINC00173 were significantly up-regulated in the AIS group, while the others were expressed in a declining tendency (Supplementary Fig. S2).

### The ceRNA network based on immune and TLR signaling pathway related feature lncRNAs

**Figure 5:**
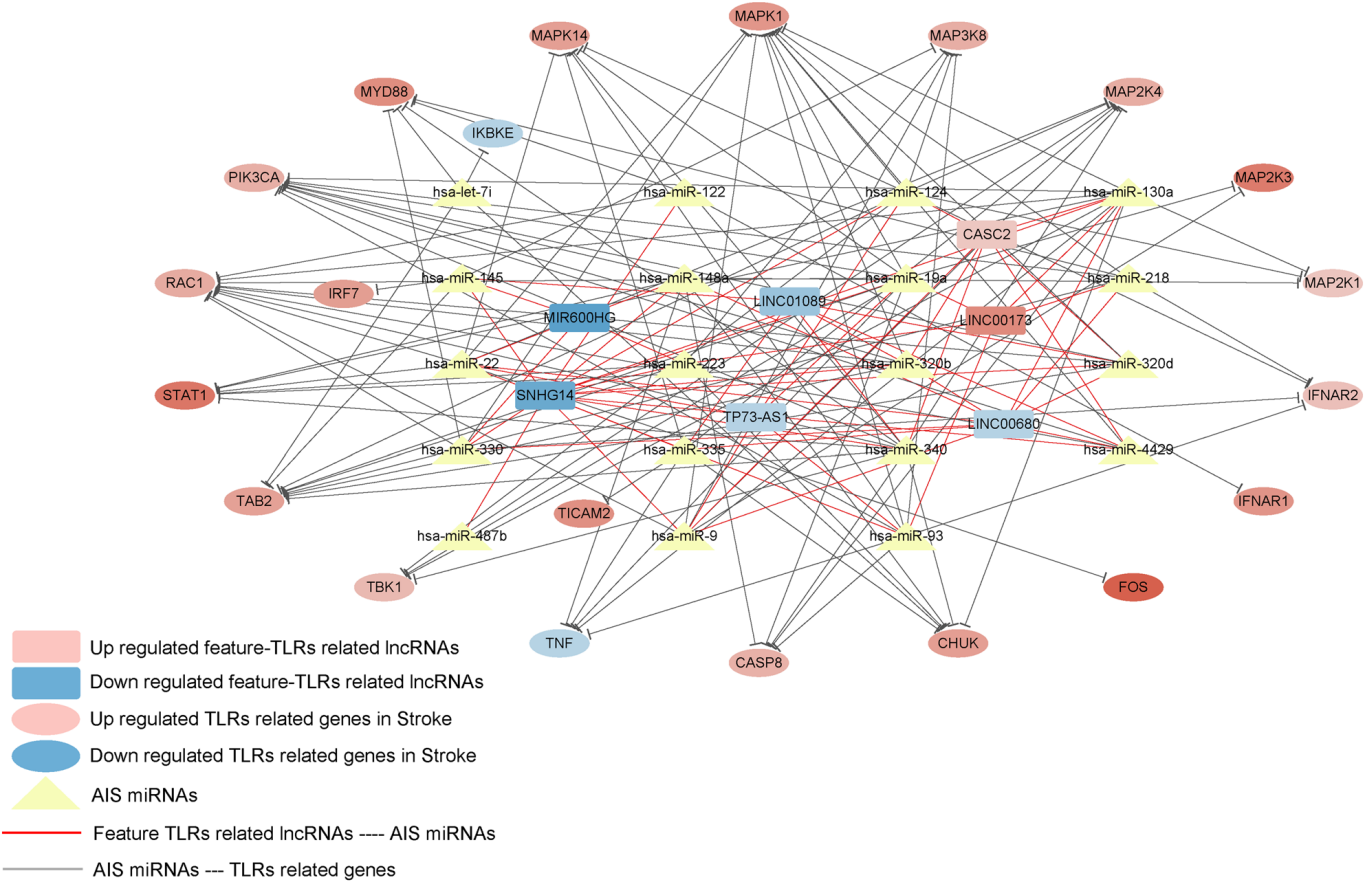
The ceRNA network consisting of the immune and TLR signaling pathway related feature lncRNAs and AIS related miRNAs and TLR signaling pathway related genes.

25 miRNAs associated with AIS were obtained from HMDD v3.2. Associations between the immune and TLR signaling pathway related feature lncRNAs and miRNAs associated with AIS were explored by DIANA-LncBasev2, and 57 associations were derived including 7 feature lncRNAs and 19 miRNAs (Supplementary Table S6). Using starBase v2.0, the mRNA targets of 19 miRNAs associated with AIS were searched. Genes that were both mRNA targets and TLR signaling pathway related DEGs were reserved, and 101 AIS related miRNA—TLR signaling pathway related gene associations were gained, including 19 miRNAs and 21 mRNAs (Supplementary Table S7). Combining the lncRNA–miRNA and miRNA–mRNA associations, a ceRNA network was constructed consisting of the immune and TLR signaling pathway related feature lncRNAs as well as AIS related miRNAs and TLR signaling pathway related genes (Fig. 5).

## Discussion

The global burden of stroke has increased in the past few years^1^. With the approved treatment of AIS are limited, new therapies are needed that can improve the clinical outcome. As transmembrane receptors, TLRs are important components of the innate immune system that mediate cerebral ischemic injury and play a crucial role in the pathogenesis of AIS^17^. Therefore, a better understanding of molecular mechanisms of TLR signaling pathway contributes to the development of immunotherapy of AIS targeting the endogenous immune response.

We applied ssGSEA for the immune infiltration analysis and found that 16 immune cell types exhibited significant differences in distribution between AIS and control groups, including activated CD8 T cell, central memory CD8 T cell, effector memory CD8 T cell, activated CD4 T cell, gamma delta T cell, regulatory T cell, activated B cell, memory B cell, natural killer cell, CD56dim natural killer cell, activated dendritic cell, plasmacytoid dendritic cell, macrophage, mast cell, monocyte and neutrophil. Gelderblom et al. also observed that T lymphocytes, neutrophils, macrophages and dendritic cells were increased in the infarcted hemisphere^18^. B cells seem to be both beneficial and detrimental in stroke^19^. The pharmacological depletion of B cells with anti-CD20, its depleting antibody, prevented the appearance of delayed cognitive deficits following stroke^20^.

The feature lncRNA selection was carried out by different optimization algorithms. Several immune and TLR signaling pathway related feature lncRNAs were obtained, including LINC00173, LINC01089, LINC02210, MIR600HG, SNHG14, TP73-AS1, LINC00680 and CASC2. Using the lncRNA signature, the diagnostic nomograms performed well in both the training set and the validation set. The knowledge of these feature lncRNA functions is poor, and previous studies linked them with cancers. For instance, LINC00173 is associated with the tumorigenesis of various cancers, including glioma, lung squamous cell carcinoma and colorectal cancer^21–23^. LINC01089 is a tumor-suppressive lncRNA in gastric cancer and lung cancer^24–26^. MIR600HG is a prognostic target of pancreatic ductal adenocarcinoma prediction, and suppresses metastasis in colorectal cancer^27,28^. TP73-AS1 regulates the progression of many tumors, such as glioblastoma and breast cancer^29,30^. CASC2 inhibits gliomas malignancy^31^. SNHG14 induces the activation of microglia in cerebral infarction^32^. However, there is lack of direct evidence regarding the practical roles of these immune and TLR signaling pathway related feature lncRNAs in AIS, which needs further investigation.

In order to demonstrate how the immune and TLR signaling pathway related feature lncRNAs act in AIS, we explored their AIS related miRNA targets and the downstream TLR signaling pathway related target genes to construct a ceRNA network. For example, SNHG14 regulates 17 miRNAs, such as miR-145, of which target genes are TAB2, IRF7 and MAP3K8. MiR-22 is modulated by 4 lncRNAs, including LINC00680, MIR600HG, TP73-AS1 and SNHG14, and has 6 target genes which are MAPK1, MAPK14, MAP2K4, FOS, STAT1 and RAC1. The regulatory network around the immune and TLR signaling pathway related feature lncRNAs is complex, however, it provides clues for the lncRNA-based immunotherapy for AIS.

There are several limitations in the present study. The datasets were limited and the sample sizes were small. Further experiments are required to explore the roles of these immune and TLR signaling pathway related feature lncRNAs and their regulatory mechanisms in the pathogenesis of AIS. Prospective researches are warranted to further demonstrate the diagnostic model in AIS patients.

## Conclusion

In conclusion, several immune and TLR signaling pathway related feature lncRNAs may be the candidate biomarkers of AIS diagnosis, including LINC00173, LINC01089, LINC02210, MIR600HG, SNHG14, TP73-AS1, LINC00680 and CASC2, providing the potential for TLR signaling pathway as the promising avenue for AIS immune therapeutic target.

## Supplementary Information

Supplementary Figure S1.

Supplementary Figure S2.

Supplementary Legends.

Supplementary Table S1.

Supplementary Table S2.

Supplementary Table S3.

Supplementary Table S4.

Supplementary Table S5.

Supplementary Table S6.

Supplementary Table S7.

## Data Availability

The raw datasets analyzed during the current study, GSE16561 and GSE22255, are available in NCBI Gene Expression Omnibus (www.ncbi.nlm.nih.gov/geo).
