# Supplementary figures and images for: Identification of immune and Toll-like receptor signaling pathway related feature lncRNAs to construct diagnostic nomograms for acute ischemic stroke
Source: Sci Rep. 2023 Apr 20;13:6492. doi: 10.1038/s41598-023-33059-5 (PMC10119310; doi:10.1038/s41598-023-33059-5)

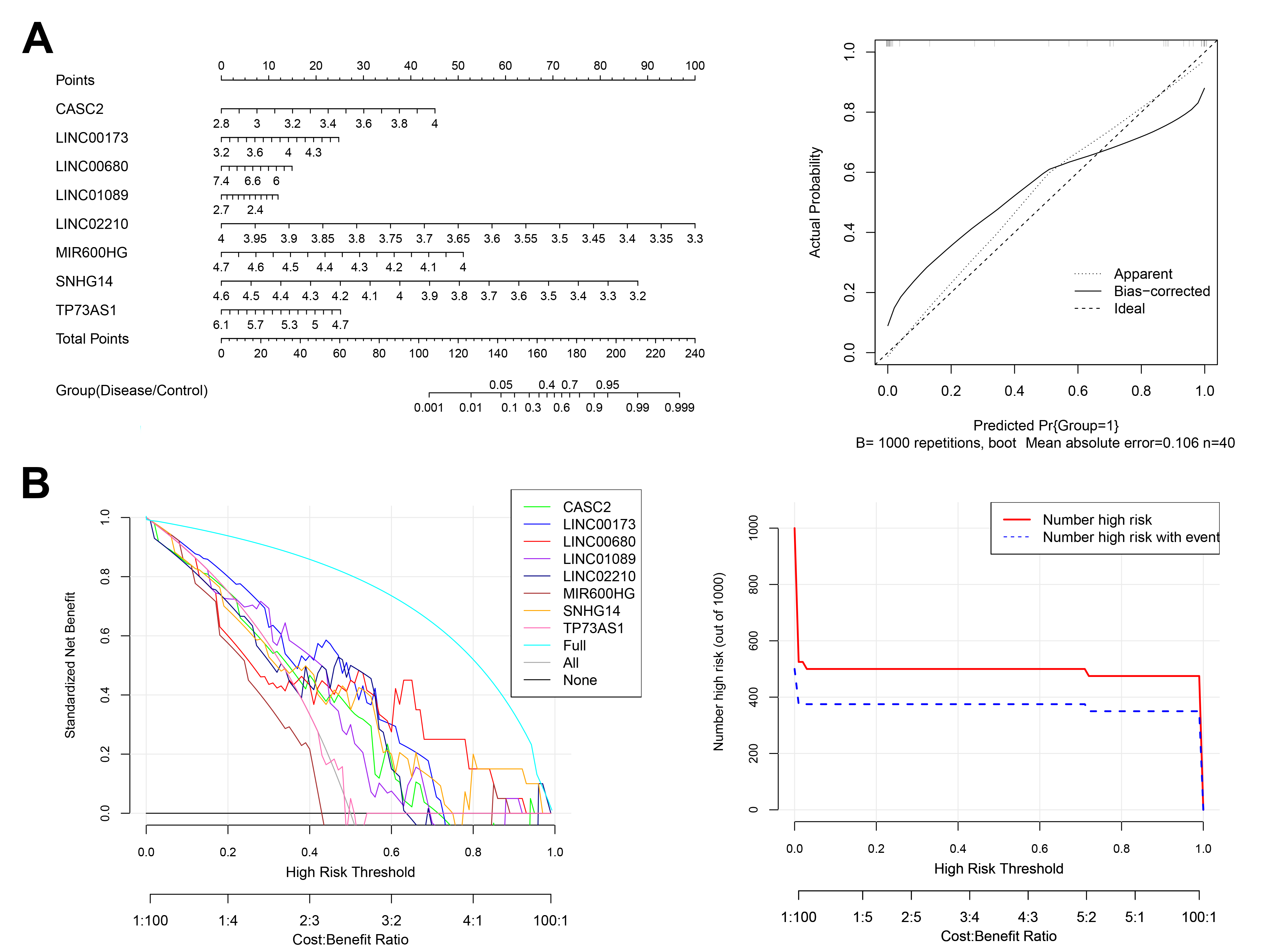

Supplement: Supplementary file 1 — Supplementary Figure S1. [file 41598_2023_33059_MOESM1_ESM.tiff]

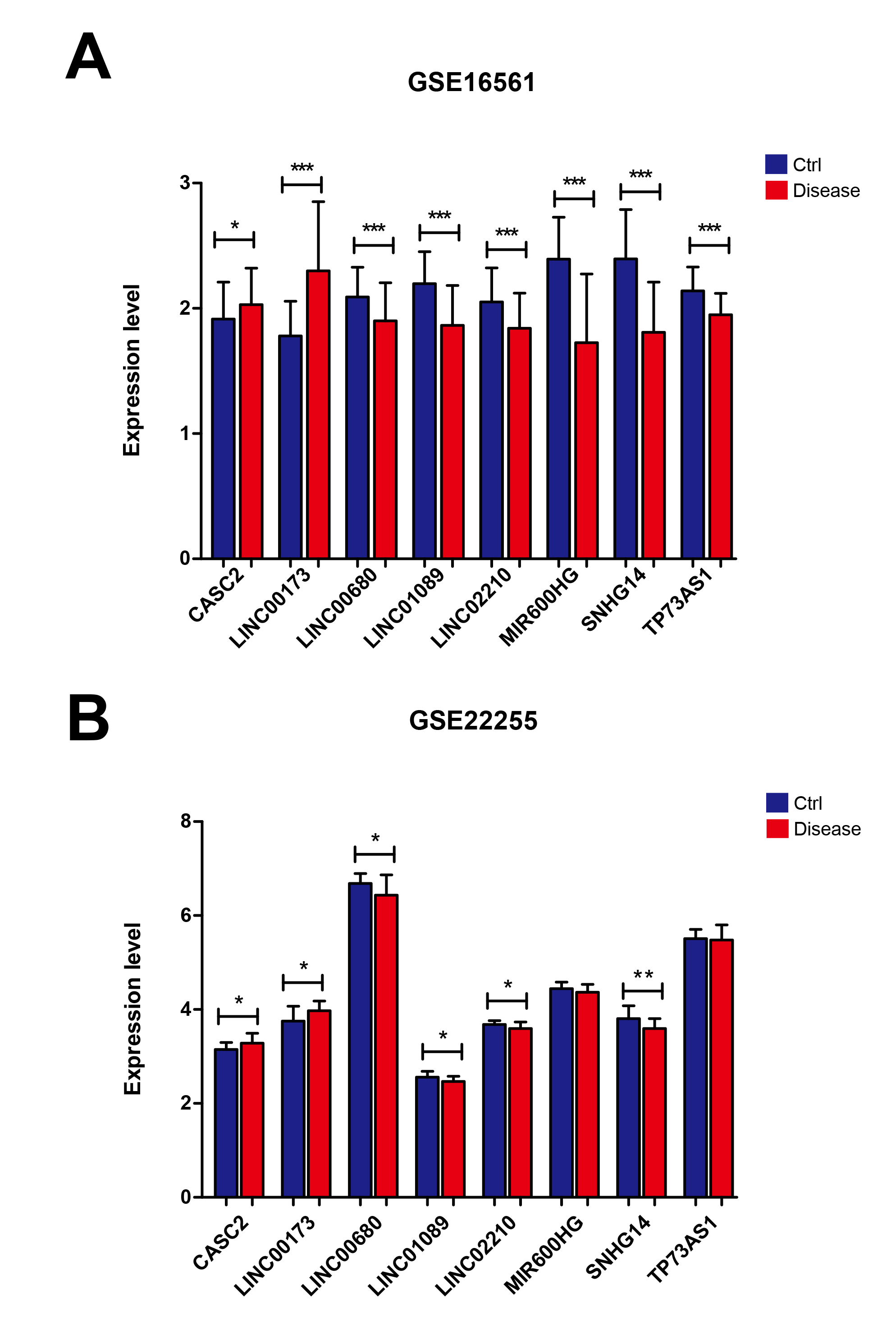

Supplement: Supplementary file 2 — Supplementary Figure S2. [file 41598_2023_33059_MOESM2_ESM.tiff]
