# Supplementary material for: Identification of immune and Toll-like receptor signaling pathway related feature lncRNAs to construct diagnostic nomograms for acute ischemic stroke
Source: Sci Rep. 2023 Apr 20;13:6492. doi: 10.1038/s41598-023-33059-5 (PMC10119310; doi:10.1038/s41598-023-33059-5)
Supplement: Supplementary file 3 — Supplementary Legends. [file 41598_2023_33059_MOESM3_ESM.docx]

Figure S1 The diagnostic nomogram applied to the validation set GSE22255 and the corresponding calibration curve (A) and the decision curve analysis (B).

Figure S2 Differential expression patterns of eight immune and TLR signaling pathway related feature lncRNAs between groups in the training set GSE16561 and the validation set GSE22255.

**Table caption**

Table S1 KEGG pathways and the corresponding gene sets.

Table S2 DElncRNAs and DEGs associated with TLR signaling pathway.

Table S3 Significant co-expression DEG-DElncRNA pairs.

Table S4 Significant lncRNA-immune cell correlations.

Table S5 Feature lncRNAs selected by LASSO, RFE and RF algorithms respectively.

Table S6 Associations between immune and TLR signaling pathway related feature lncRNAs and AIS related miRNAs.

Table S7 Associations between AIS related miRNAs and TLR signaling pathway related genes.
